# Effects of Alcohol Dependence and Withdrawal on Stress Responsiveness and Alcohol Consumption

**DOI:** 10.35946/arcr.v34.4.09

**Published:** 2012

**Authors:** Howard C. Becker

**Affiliations:** **Howard C. Becker, Ph.D.,***is a professor of psychiatry and neuroscience at the Charleston Alcohol Research Center, Department of Psychiatry and Behavioral Sciences, Department of Neurosciences, Medical University of South Carolina, and a medical research career scientist at the Ralph H. Johnson Veterans Affairs Medical Center, both in Charleston, South Carolina.*

**Keywords:** Alcohol consumption, alcohol dependence, chronic alcohol exposure, drinking behavior, withdrawal, relapse, stress, stress response, biological adaptation to stress, brain, brain stress pathway, hypothalamic, pituitary, adrenocortical axis, corticotropin-releasing factor, corticosteroids, norepinephrine, human studies, animal models

## Abstract

A complex relationship exists between alcohol-drinking behavior and stress. Alcohol has anxiety-reducing properties and can relieve stress, while at the same time acting as a stressor and activating the body’s stress response systems. In particular, chronic alcohol exposure and withdrawal can profoundly disturb the function of the body’s neuroendocrine stress response system, the hypothalamic–pituitary–adrenocortical (HPA) axis. A hormone, corticotropin-releasing factor (CRF), which is produced and released from the hypothalamus and activates the pituitary in response to stress, plays a central role in the relationship between stress and alcohol dependence and withdrawal. Chronic alcohol exposure and withdrawal lead to changes in CRF activity both within the HPA axis and in extrahypothalamic brain sites. This may mediate the emergence of certain withdrawal symptoms, which in turn influence the susceptibility to relapse. Alcohol-related dysregulation of the HPA axis and altered CRF activity within brain stress–reward circuitry also may play a role in the escalation of alcohol consumption in alcohol-dependent individuals. Numerous mechanisms have been suggested to contribute to the relationship between alcohol dependence, stress, and drinking behavior. These include the stress hormones released by the adrenal glands in response to HPA axis activation (i.e., corticosteroids), neuromodulators known as neuroactive steroids, CRF, the neurotransmitter norepinephrine, and other stress-related molecules.

Although stress is known to be an important contributing factor to alcohol abuse and alcoholism, the interaction between stress and alcohol drinking behavior, as well as the mechanisms underlying this interaction in the context of dependence are complex and not well understood. On the one hand, alcohol is an effective anxiety-reducing agent (i.e., anxiolytic). Hence, motivation for drinking may be related to its ability to alleviate stress, including stress associated with periods of abstinence following bouts of heavy drinking (Cappell and Greeley 1987; Sayette 1999). On the other hand, alcohol itself can serve as a stressor, activating the hypothalamic–pituitary–adrenocortical (HPA) axis, which constitutes a major component of the hormonal (i.e., neuroendocrine) stress response (Smith and Vale 2006). Furthermore, chronic alcohol exposure and withdrawal experiences not only produce robust perturbations in the HPA axis but also engage neuroendocrine-independent (i.e., extrahypothalamic) brain stress systems that influence drinking behavior in a dynamic and complex manner (Koob and Kreek 2007).

The relationship between stress and alcohol drinking is complicated by a host of alcohol-related factors (e.g., history of use, level and pattern of drinking, or timing of accessibility of alcohol in relation to stress experience) as well as stress-related factors (e.g., type, chronicity, intermittency, predictability, and controllability) that intersect with a number of biological variables (e.g., genetics, age, and sex). For example, clear individual differences exist in sensitivity to, perception of, and responsiveness to stress and alcohol, and both clinical and preclinical evidence indicate that genetic factors help shape the nature of the relationship between stress and alcohol drinking (Clarke et al. 2008; Uhart and Wand 2009). The dynamic interaction of these biological and environmental variables along with experiential factors plays a critical role in defining subjective aspects of stress (i.e., the perception and appraisal of a stressful event) and alcohol intoxication. These subjective effects, in turn, shape the impact of stress on alcohol drinking and of alcohol consumption on stress responsiveness.

Despite the complex interaction between stress and alcohol consumption, it generally is acknowledged that stressful life events prominently influence alcohol drinking and, in particular, relapse (Brady and Sonne 1999; Sinha 2001, 2008). Several animal models have been developed to study the influence of stress on alcohol consumption. However, reviews of this literature have found equivocal results regarding the circumstances and manner in which stress modulates alcohol drinking (Becker et al. 2011; Pohorecky 1990; Sillaber and Henniger 2004). The discrepancies in results no doubt relate to the aforementioned plethora of variables that influence the reciprocal relationship between stress and alcohol. Nevertheless, researchers continue to focus on stress associated with chronic alcohol exposure and withdrawal experiences and recently have directed attention to stress–alcohol interactions in alcohol-dependent subjects (Becker et al. 2011; Heilig et al. 2010; Pohorecky 1990; Sillaber and Henniger 2004).

This article provides an overview of clinical studies and studies involving animal models of alcohol dependence that demonstrate both prolonged alcohol exposure and repeated periods of abstinence constitute potent stressors to the organism. Studies conducted in rodents, monkeys, and humans are described that highlight the impact of chronic alcohol exposure and withdrawal on neuroendocrine and brain stress pathways, as well as how activation of these brain stress systems, which are closely linked to brain reward systems, alter motivation to drink. Finally, evidence will be presented that stress associated with alcohol dependence not only compromises the ability to mount an appropriate behavioral response to a subsequent stress challenge, but also alters the ability of stress challenges to modulate drinking in the dependent state.

## Stress Associated With Chronic Alcohol Exposure and Withdrawal

As previously noted, alcohol activates the HPA axis, with the magnitude and response profile influenced by a host of variables, including the individual’s genetic makeup (i.e., genotype) and sex as well as dosing parameters (Rivier 2000; Wand 2000). Alcohol stimulates neuronal activity in the paraventricular nucleus of the hypothalamus, thereby inducing release of corticotropin-releasing factor (CRF) (and vasopressin) from these cells. CRF, in turn, induces the secretion of adrenocorticotrophic hormone (ACTH) from the pituitary, which subsequently acts on the adrenal glands to cause an increase in the circulating levels of glucocorticoids (e.g., cortisol in humans and corticosterone in rodents) (Lee et al. 2001, 2004).

Both clinical and experimental studies have documented profound disturbances in HPA axis function following chronic alcohol exposure and withdrawal. For example, studies in humans (Errico et al. 1993; Wand and Dobs 1991), monkeys (Helms et al. 2012*a*, *b*), and rodents (Kakihana and Moore 1976; Lee et al. 2000; Rasmussen et al. 2000; Tabakoff et al. 1978) have shown that chronic alcohol consumption produces general elevation in blood glucocorticoid levels, flattening of normal circadian fluctuations, and a dampened HPA response to subsequent stress challenge. Periods of abstinence (i.e., withdrawal) also are characterized by elevated glucocorticoid levels that reflect increased HPA axis activity, as well as by increased activity of the sympathetic division of the autonomic nervous system^1^ that produces an array of physiological symptoms, including rapid heartbeat (i.e., tachycardia), elevated blood pressure (i.e., arterial hypertension), excessive sweating (i.e., diaphoresis), and body temperature dysregulation (Becker 2000; Heilig et al. 2010). For example, studies in rats have demonstrated increased activity of the adrenal glands and sympathetic nervous system (i.e., sympathoadrenal activity) during alcohol withdrawal, as evidenced by elevated plasma levels of the epinephrine and norepinephrine^2^ (Rasmussen et al. 2006). Similarly, increased concentrations of norepinephrine in cerebrospinal fluid were reported during acute alcohol withdrawal in alcoholics (Hawley et al. 1994). Finally, elevated plasma levels of epinephrine (Ehrenreich et al. 1997) and norepinephrine (Patkar et al. 2003, 2004) have been reported in abstinent alcoholics.

As is the case with most physiological features of alcohol withdrawal, autonomic-related symptoms typically wax and wane over the course of acute withdrawal; however, some cardiovascular changes may persist, especially when assessed following a stress challenge (Bernardy et al. 2003; Kahkonen 2004; King et al. 1996). Likewise, studies in humans and animals have shown that whereas heightened HPA axis activation associated with withdrawal usually resolves within a few days (Adinoff et al. 1991; Tabakoff et al. 1978), the blunted HPA axis responsiveness, along with reduced basal levels of circulating corticosteroids, appear to persist for a protracted period of time (Adinoff et al. 1990; Cuzon Carlson et al. 2011; Lovallo et al. 2000; Rasmussen et al. 2000; Zorrilla et al. 2001).

In addition to these HPA-axis–related effects, alcohol alters the activity of the stress-related neuropeptide CRF outside of the HPA axis (Heilig and Koob 2007; Koob and Zorrilla 2010; Uhart and Wand 2009). Increased CRF activity in several brain structures following chronic alcohol exposure represents an important neuroadaptive change that is thought to be key in the emergence of withdrawal-related anxiety and dysphoria, which likely are intimately tied to alcohol drinking and relapse (Becker 2009; Heilig et al. 2010; Heilig and Koob 2007; Koob and Kreek 2007). Moreover, there is evidence that norepinephrine and CRF systems in the brain not only interact closely to mediate behavioral responses to stress, but also play an important role in negative affective states and relapse vulnerability during alcohol/drug abstinence (Dunn and Swiergiel 2008; Smith and Aston-Jones 2008). Thus, chronic alcohol exposure and withdrawal experiences can be viewed as potent stressors that disrupt the functional integrity of the HPA axis as well as recruit extrahypothalamic CRF and other brain stress systems. This perturbation in brain and neuroendocrine stress systems may have significant implications regarding motivation for alcohol self-administration.

## Role of CRF in Stress Associated With Alcohol Dependence and Withdrawal

CRF is a 41 amino-acid neuropeptide that is distributed widely throughout the mammalian brain. It is found in high concentrations in the paraventricular nucleus of the hypothalamus where it acts to regulate HPA axis activity, which is critical for orchestrating behavioral and physiological responses to stress. CRF-containing neurons also are found in many brain regions outside the HPA axis, including an extensive network of interconnected neural structures (e.g., amygdala, bed nucleus of the stria terminals, and prefrontal cortex) that are intimately associated with the brain’s reward and stress pathways. The actions of CRF (and of the related peptides urocortin I, II, and III) are modulated by CRF-binding protein and mediated through interaction with two receptors known as excitatory G-protein-coupled receptors (i.e., CRF_1_ and CRF_2_ receptors) (Bale and Vale 2004). These receptors are distributed in overlapping yet distinct patterns within the brain’s reward and stress circuits. This anatomical distribution of CRF and its associated binding sites is congruent with the importance of both hypothalamic and extra-hypothalamic CRF in processing and regulating central, autonomic, and emotional/behavioral responses to stress as well as to rewarding stimuli/events, including alcohol and other drugs of abuse (Bruijnzeel and Gold 2005; Ryabinin et al. 2002).

A large body of evidence indicates that CRF plays a significant role in alcohol (and other drug) addiction (Heilig and Koob 2007; Koob and Zorrilla 2010; Lowery and Thiele 2010). Chronic alcohol exposure can alter CRF neurotransmission as evidenced by withdrawal-related HPA axis activation and long-lasting dysregulation (Adinoff et al. 1990; Rivier 2000). In addition, time-dependent changes in extracellular levels of extra-hypothalamic CRF occur during withdrawal (Merlo Pich et al. 1995; Olive et al. 2002; Zorrilla et al. 2001). Numerous studies have shown that such changes in brain CRF activity have important ramifications regarding alcohol self-administration. For example, CRF infusion into the brain ventricles^3^ reduces voluntary alcohol intake in rats (Bell et al. 1998; Thorsell et al. 2005). Likewise, mice genetically engineered to produce higher-than-normal CRF levels (i.e., CRF transgenic mice) exhibited reduced voluntary alcohol intake compared with nontransgenic control animals (Palmer et al. 2004), whereas CRF-deficient mice showed the opposite effect (i.e., increased alcohol drinking) (Olive et al. 2003). Also, there is evidence that basal differences in brain CRF expression may relate to genetically determined differences in the propensity to drink (Ehlers et al. 1992; Hayes et al. 2005).

Indeed, a strong genetic influence on stress–alcohol interactions is related to the role of CRF in mediating stress responsiveness as well as alcohol drinking and risk for dependence. Recent studies in humans, monkeys, and rats have suggested that an association exists between certain gene variants involving only a single DNA building block (i.e., single nucleotide polymorphisms [SNPs]) of the CRF and CRF_1_ receptor genes and alcohol drinking (Barr et al. 2008, 2009; Blomeyer et al. 2008; Chen et al. 2010; Schmid et al. 2010). For example, studies in rhesus macaque monkeys have shown that SNPs in various components of the regulatory region (i.e., promoter) for the gene encoding CRF (i.e., the *Crh* gene) affected several stress- and alcohol-related behaviors. Thus, a SNP in the glucocorticoid response element region of the *Crh* promoter (Crh-2232 C➝G) predicted bold behavior and high-risk drinking, whereas a SNP in the cAMP response element region of the *Crh* promoter (Crh-248 C➝G) conferred augmented stress reactivity and elevated alcohol drinking, but only with a history of early stress/trauma (Barr et al. 2008, 2009). In a longitudinal human study, a history of early childhood stress/trauma events interacted with two SNPs in the gene encoding the CRF_1_ receptor (i.e., the *CrhR1* gene) that were associated with earlier age for drinking onset as well as heavier drinking at young adulthood (Blomeyer et al. 2008; Schmid et al. 2010). In another clinical study, several other SNPs in the *CrhR1* gene were associated with the height (i.e., amplitude) of a component P3 of a brainwave known as an event-related potential (ERP)^4^ as well as with an alcohol dependence diagnosis (Chen et al. 2010).

Additional evidence for the relationship between genetic variation in the *CrhR1* gene and vulnerability to alcoholism comes from a study in rats (Hansson et al. 2006). These investigators examined the relationship of *CrhR1* expression in brain and stress reactivity as well as the ability of stress to reinstate alcohol-seeking behavior in rats that were selectively bred for high alcohol preference over many generations (i.e., Marchigian-Sardinian Preferring [msP] rats) and in control rats (i.e., outbred Wistar rats). The msP rats showed elevated *CrhR1* expression in several limbic brain regions (e.g., several sub-regions of amygdala and hippocampus) as well as greater behavioral stress reactivity and greater sensitivity to stress-induced reinstatement of alcohol responding. This latter effect was blocked by an agent that can interfere with the activity of the CRF_1_ receptor (i.e., the CRF_1_ receptor antagonist antalarmin) in msP rats but not Wistar rats. Also, a sequence variation in the promoter region of *CrhR1* was more commonly found in msP rats compared with the control rats. Collectively, these findings indicate that genetic variations in the *Crh* and the *CrhR1* genes interact with stressful life events to influence age of drinking onset, progression of heavy drinking in adulthood, and general vulnerability to alcohol dependence.

Changes in CRF activity resulting from chronic alcohol exposure appear to be key to the emergence of affective-related withdrawal symptoms that may be especially relevant in promoting excessive drinking and enhanced susceptibility to relapse. For example, increased anxiety associated with alcohol withdrawal is reduced by administration of non-selective CRF receptor antagonists into the ventricles (Baldwin et al. 1991; Valdez et al. 2003) or the central nucleus of the amygdala (Rassnick et al. 1993). Selective CRF_1_ receptor antagonists administered not directly into the brain (i.e., systemically) produced similar effects, suggesting that withdrawal-related anxiety is mediated by CRF_1_ receptors (Breese et al. 2005; Sommer et al. 2008), although a role for CRF_2_ receptors cannot be ruled out (Valdez et al. 2004).

Studies using operant reinstatement procedures also have demonstrated an important role for CRF in mediating the ability of stress to trigger relapse-like behavior. For example, CRF antagonists can prevent stress-induced increases in alcohol-seeking behavior (Gehlert et al. 2007; Le et al. 2000; Liu and Weiss 2002; Marinelli et al. 2007). This effect appears to be mediated by extra-hypothalamic CRF activity, because removal of the adrenal glands (i.e., adrenalectomy) with or without corticosterone supplementation did not affect reinstatement of alcohol responding induced by foot-shock stress (Le et al. 2000). Direct infusion of a CRF antagonist into a brain structure, the median raphe nucleus, blocked stress-induced alcohol seeking behavior (Le et al. 2002). Taken together, this body of evidence suggests that stress associated with alcohol dependence produces significant changes in CRF function within the brain and neuroendocrine systems that may directly, and/or by mediating withdrawal-related anxiety and stress/dysphoria responses, influence motivation to engage in alcohol self-administration.

## Alcohol Dependence, Stress, and Drinking

Alcohol dependence long has been postulated to play a significant role in driving and maintaining excessive drinking. Numerous studies involving rodents have demonstrated that alcohol-dependent animals consume increasing amounts of alcohol if they are given free choice between water and an alcohol solution or if they are rewarded with alcohol after performing a certain task (i.e., in operant conditioning procedures). In most cases, dependence has been induced by delivering alcohol vapor via inhalation chambers. For example, one mouse model of dependence and relapse drinking has demonstrated that repeated cycles of chronic alcohol exposure delivered by inhalation result in an escalation of voluntary alcohol drinking (Becker and Lopez 2004; Lopez and Becker 2005). More detailed analysis of the pattern of alcohol consumption revealed that dependent mice not only consumed greater overall amounts of alcohol compared to non-dependent mice, but also the rate of consumption was faster and progressively increased over successive withdrawal test periods (Griffin et al. 2009*b*). This escalation of alcohol consumption in dependent mice produced significantly higher and more sustained blood and brain alcohol levels compared with that achieved by more modest (stable) intake in nondependent mice (Griffin et al. 2009*b*). Additionally, increased numbers of cycles of chronic intermittent alcohol exposure resulted in greater and longer lasting enhancement of voluntary alcohol drinking (Griffin et al. 2009*a*; Lopez and Becker 2005). Importantly, this effect appeared specific to alcohol because the animals exhibited no changes in water intake or consumption of palatable fluids, including sucrose and saccharin solutions (Becker and Lopez 2004; Lopez et al. 2012). Other investigators have reported similar results using inhalation procedures in mice (Dhaher et al. 2008; Finn et al. 2007) and rats (Rimondini et al. 2002; Sommer et al. 2008). Likewise, studies using operant procedures have demonstrated increased alcohol self-administration in mice (Chu et al. 2007; Lopez et al. 2006) and rats (Gilpin et al. 2009; O’Dell et al. 2004*b*; Roberts et al. 2000) with a history of repeated chronic intermittent alcohol exposure. Additional evidence indicates that repeated alcohol exposure enhances the reinforcing efficacy of alcohol (Brown et al. 1998; Lopez et al. 2008). Studies in mice and rats further have demonstrated that significant escalation of alcohol self-administration is facilitated when chronic alcohol vapor exposure to induce dependence occurs intermittently rather than continuously (Lopez and Becker 2005; O’Dell et al. 2004*b*). These latter findings suggest that stress associated with chronic alcohol exposure and, in particular, repeated experience with alcohol withdrawal is crucial for the enhanced motivation to consume alcohol.

Indeed, several studies have demonstrated that dependence models involving chronic intermittent alcohol exposure constitute potent stressors, as evidenced by initial activation and subsequent dysregulation of HPA axis activity (Lopez et al. 2010; Richardson et al. 2008). More specifically, increased cycles of chronic intermittent alcohol exposure appeared to blunt HPA axis activation, as measured by reduced levels of plasma corticosterone (Lopez et al. 2010). This reduced HPA response was observed just prior to withdrawal and at peak withdrawal in a mouse model of alcohol dependence. Recent studies suggest that this dampening of HPA axis activity may relate to enhanced activity of receptors for the neurotransmitter γ-aminobutyric acid (i.e., increased GABA_A_ receptor function) (Li et al. 2011) and/or reduced number of CRF-releasing neurons (Silva et al. 2009) in the paraventricular nucleus of the hypothalamus. These stress-related adaptations produced by chronic alcohol exposure and withdrawal may underlie the long-lasting dampening of basal and stress-stimulated HPA axis activity that has been observed in abstinent alcoholics (Adinoff et al. 1990; Lovallo et al. 2000; Rasmussen et al. 2000).

In addition to engendering elevated drinking and perturbations in HPA axis function, prolonged alcohol exposure also enhances behavioral responsiveness to stress. For example, rats exhibit increased stress responsiveness following withdrawal from chronic alcohol exposure, as measured by several experimental procedures that provoke behavioral measures of stress/anxiety, such as reduced social interaction in a novel environment, reduced exploration in threatening circumstances (e.g., open, brightly illuminated spaces), and greater electroshock-induced suppression of ongoing behavior (Breese et al. 2005; Gehlert et al. 2007; Sommer et al. 2008). Thus, whereas prolonged alcohol exposure and withdrawal experiences lead to disturbances in homeostatic regulation of HPA axis function, behavioral sensitization to stress may be critical in rendering subjects more vulnerable to relapse and return to uncontrolled, harmful levels of alcohol consumption. Indeed, experimental evidence suggests that stress can provoke relapse-like behavior and increase alcohol drinking more easily in subjects with a history of dependence (Liu and Weiss 2002; Sommer et al. 2008).

### Mechanisms Underlying the Alcohol Dependence–Stress–Drinking Relationship

The mechanisms by which stress associated with chronic alcohol exposure and withdrawal influences excessive drinking and increased relapse vulnerability are not fully understood, but several pathways have been suggested.

#### Role of Corticosteroids

Elevated glucocorticoid levels resulting from dependence-related HPA axis activation may contribute to amplified motivation to drink through an interaction with the brain’s reward system, the mesocorticolimbic reward circuitry (Piazza and Le Moal 1997). Central and systemic administration of corticosterone has been shown to increase alcohol consumption, whereas adrenalectomy or administration of a corticosteroid synthesis inhibitor (i.e., metyrapone) decreased alcohol intake in rodents (Fahlke et al. 1995, 1996). Likewise, a glucocorticoid receptor antagonist (i.e., mifepristone) reduced alcohol self-administration behavior (Koenig and Olive 2004). Furthermore, mifepristone administered systemically or into the central nucleus (but not the basolateral nucleus) of the amygdala attenuated stress-induced reinstatement of alcohol seeking behavior (Simms et al. 2012).

Chronic corticosterone exposure in rats also can reduce sensitivity to the subjective (i.e., discriminative stimulus) effects of alcohol (Besheer et al. 2012). A similar outcome also has been reported following chronic alcohol exposure and withdrawal in mice (Becker and Baros 2006). These results suggest that following chronic alcohol exposure and withdrawal, blunted subjective feedback regarding intoxication (possibly related to changes in HPA axis activity) may act as a permissive factor promoting higher levels of drinking. Studies in mice and rats also have shown that withdrawal following prolonged alcohol consumption produced elevated corticosterone levels in certain brain regions (i.e., the prefrontal cortex and hippocampus) that persisted long after plasma corticosterone levels returned to baseline levels (Little et al. 2008). Elevations in brain glucocorticoid concentrations following chronic alcohol exposure and withdrawal not only may have significant implications for motivation to drink, but also may contribute to the cognitive deficits and neurotoxic damage that is commonly associated with alcohol dependence (Rose et al. 2010).

#### Role of Neuroactive Steroids

HPA axis activity also can influence brain activity through the actions of molecules known as neuroactive steroids. Neuroactive steroids are endogenous neuromodulators that interact with several neurotransmitter systems via rapid membrane action (as opposed to other steroid molecules that act via slower intracellular genomic mechanisms) (Genazzani et al. 1998; Patchev et al. 1994, 1996). Among the neuroactive steroids, compounds 3α,5α-THDOC and 3α,5α-THP, or allopregnanolone, which are the 3α,5α-reduced metabolites of deoxycorticosterone and progesterone, respectively, are the most potent positive modulators of GABA_A_ receptors. These compounds produce anxiolytic, anticonvulsant, and sedative/hypnotic effects similar to other positive modulators of the GABA_A_ receptor, including alcohol (Khisti et al. 2002; Morrow et al. 2001; Rupprecht and Holsboer 1999). Additionally, these neuroactive steroids can modulate a variety of alcohol effects, including anticonvulsant, anxiolytic, ataxic/sedative, and cognitive-impairing effects, as well as the discriminative stimulus and reinforcing effects of alcohol (Khisti et al. 2002; Morrow et al. 2001).

Both alcohol and stress increase plasma and brain concentrations of neuroactive steroids in rodents (Barbaccia et al. 1999, 2001; Finn et al. 2010). This increase appears to be mediated by activation of the HPA axis because the increase in neuroactive steroid levels elicited by these stimuli can be blocked by disruption of the HPA axis via adrenalectomy (O’Dell et al. 2004*a*; Purdy et al. 1991). Alcohol and stress also have been reported to produce elevations in plasma concentrations of neuroactive steroids in humans, but the effects are not entirely consistent (Holdstock et al. 2006; Pierucci-Lagha et al. 2006; Torres and Ortega 2003, 2004). Chronic alcohol exposure also can alter brain and plasma levels of neuroactive steroids in rodents and humans (Cagetti et al. 2004; Janis et al. 1998; Morrow et al. 2009; Romeo et al. 1996). Such neuroadaptive changes in activity of neuroactive steroids may enhance the motivational effects of alcohol, perhaps by modifying the expression and/or function of GABA_A_ receptors (Biggio et al. 2007; Finn et al. 2010; Morrow et al. 2001; Purdy et al. 2005) and/or through interactions with CRF (Genazzani et al. 1998; Patchev et al. 1994, 1996). In fact, in a mouse model of chronic intermittent alcohol exposure and withdrawal, increased drinking was accompanied by increased expression of allopregnanolone in the brain (Morrow et al. 2009).

Additional evidence suggests that changes in activity of neuroactive steroids play a role in dependence, especially in the expression of withdrawal symptoms as well as alcohol drinking (Finn et al. 2010). For example, allopregnanolone administered systemically (Ford et al. 2005; Sinnott et al. 2002) or directly into the brain or ventricles (Finn et al. 2007; Janak and Gill 2003; Janak et al. 1998) altered alcohol self-administration in male rodents in a dose-dependent manner, with low doses increasing intake and higher doses reducing consumption. In contrast, female animals were relatively insensitive to this biphasic effect of allopregnanlone (Ford et al. 2008), possibly because they have higher basal levels of allopregnanolone (Finn et al. 2010). Finally, allopregnanolone can induce relapse-like behavior in mice (Finn et al. 2008) and rats (Nie and Janak 2003).

#### Role of CRF

As noted above, numerous studies have demonstrated a significant role for altered CRF activity in dependence-related alcohol drinking. The mouse model of dependence and relapse drinking described earlier has provided evidence for reduced HPA axis activation and compromised behavioral response to a stress challenge. At the same time, additional findings point to an accentuation of changes in the expression and release of CRF in extrahypothalamic brain regions that are implicated in motivational effects of alcohol (Doremus-Fitzwater and Becker 2010; Griffin et al. 2011; Lopez et al. 2010). The role of CRF further is emphasized by observations that a nonselective peptide CRF antagonist (i.e., D-Phe-CRF_12–41_) reduced excessive drinking in dependent animals when administered into the brain ventricles (Funk et al. 2007; Valdez et al. 2002) or into the central nucleus of the amygdala (Funk et al. 2006*a*, *b*). Further, systemic administration of selective antagonists for the CRF_1_ receptor reduced upregulated drinking in dependent mice (Chu et al. 2007) and rats (Funk et al. 2007; Gehlert et al. 2007; Gilpin et al. 2008*a*; Roberto et al. 2010; Sommer et al. 2008).

#### Role of Norepinephrine

Stress associated with alcohol dependence also includes activation of the locus coeruleus, a nucleus of cells in the brainstem that provides most of the norepinephrine in the brain. This increase in noradrenergic activity plays a role in mediating both somatic and affective aspects of alcohol withdrawal. For example, studies in animal models and clinical investigations have demonstrated that reducing the overall level of noradrenergic activity by stimulating presynaptic autoreceptors with alpha2-adrenergic agonists (e.g., clonidine, dexmedetomidine) is effective in ameliorating various symptoms associated with the excessive activation of the sympathetic nervous system that is characteristic of withdrawal. Therefore, this pharmacological approach may be useful as an adjunct in the management of alcohol detoxification (Muzyk et al. 2011). Additional evidence suggests that alcohol dependence–related changes in brain norepinephrine activity might influence motivation to drink. When investigators reduced norepinephrine activity in the brain by blocking certain norepinephrine receptors (i.e., postsynaptic alpha1-adrenergic receptors) with an antagonist, prazosin, alcohol consumption was reduced in both dependent rats (Walker et al. 2008) and alcohol-dependent humans (Simpson et al. 2009). Likewise, treatment with antagonists (e.g., propranolol) for another type of norepinephrine receptor (i.e., the beta-adrenoceptor) also reduced drinking in dependent rats (Gilpin and Koob 2010).

#### Roles of Other Stress-Related Molecules

Studies using animal models of dependence and withdrawal also have shown that various other stress-related neuropeptides and modulators within the brain’s stress–reward pathways may help drive and/or mediate excessive levels of alcohol drinking. For example, a molecule, neuropeptide Y (NPY), is thought to serve as an anti-stress mediator, in many cases having opposite effects to CRF in the brain (Heilig et al. 1994). Likewise, neuromodulators known as endogenous opioids play a role in mediating and regulating endocrine, autonomic, and behavioral responses to stress (Drolet et al. 2001). Both the NPY system (Gilpin et al. 2011; Thorsell et al. 2005*a*) and the opioid system (Gilpin et al. 2008*a*; Walker et al. 2011) have been implicated in excessive drinking following chronic intermittent alcohol exposure. A compound, brain-derived neurotrophic factor (BDNF), also has been implicated in stress and addiction processes (Briand and Blendy 2010; Chourbaji et al. 2011; Davis 2008). Thus, regional changes in BDNF expression and/or activity in the brain following chronic alcohol exposure may play a role in mediating withdrawal-related anxiety and regulation of alcohol consumption (Logrip et al. 2009; Pandey et al. 2006). Finally, other stress-responsive systems (e.g., adrenergic, Substance P, and orexin/hypocretin systems) have been shown to influence alcohol consumption (Ciccocioppo et al. 2009; Heilig et al. 2010; Sinha et al. 2011), but their role in mediating excessive drinking associated with dependence has not been specifically examined.

## Summary

The bidirectional relationship between alcohol consumption, particularly alcohol dependence and withdrawal, and stress is complex. Clinical and preclinical evidence indicates that chronic alcohol use and withdrawal experience constitute potent stressors, leading to HPA axis activation and long-lasting dysregulation of the neuroendocrine stress response as well as perturbations in sympathetic nervous system activity. In addition, extrahypothalamic CRF activity is altered following chronic alcohol exposure and withdrawal, which in turn influences motivation to drink as well as relapse vulnerability. These observations point to a central role of CRF in the alcohol dependence–stress relationship. This pivotal role further is supported by findings that genetic variations in genes encoding CRF and its receptors can influence susceptibility to alcohol dependence as well as a variety of stress- and alcohol-related behaviors.

In addition, changes in CRF activity, both in the context of the HPA axis and in extrahypothalamic circuitry, have been related to the development of withdrawal symptoms and to the ability of stress to trigger relapse and alcohol-seeking behavior. Indeed, research has demonstrated that a history of dependence not only promotes escalation of alcohol consumption, but prolonged alcohol exposure and withdrawal experience also result in enhanced responsiveness to stress. This enhanced behavioral sensitivity to stress may increase an individual’s vulnerability to relapse, particularly in stressful situations, and further exacerbate heavy drinking associated with dependence.

In order to better understand and, ultimately, be able to disrupt the detrimental relationship between alcohol consumption, dependence, and stress, researchers are seeking to elucidate the mechanisms underlying these complex relationships. These investigations have demonstrated that in addition to the impact that CRF has on the alcohol dependence–stress relationship, other factors, such as corticosteroids, neuractive steroids, norepinephrine, and other stress-related molecules all are contributing factors. Clearly, more experimental work focused on identifying neuroadaptive changes within relevant motivational and stress pathways associated with dependence that promote/mediate excessive drinking is key to better understanding the complex reciprocal relationship between stress and alcohol, and conditions in which stress modulates drinking in the context of dependence.

## Footnotes

1 The autonomic nervous system controls involuntary functions of many internal organs. It can be divided into the sympathetic nervous system, which promotes actions requiring quick responses (i.e., the fight-or-flight response), and the parasympathetic nervous system, which promotes responses that do not require immediate action (i.e., the rest-and-digest response).

2 Epinephrine and norepinephrine (also known as adrenaline and noradrenaline) are two hormones and neurotransmitters that are produced in some nerve cells (i.e., neurons) as well as in the adrenal glands and which have many functions in the body. They are both part of the fight-or-flight response of the sympathetic nervous system.

3 The ventricles are large cavities in the brain filled with cerebrospinal fluid, which bathes the central nervous system and plays a crucial role in maintaining a stable environment for the brain.

4 ERPs are spikes in brain activity that occur in response to a specific signal, and the P3 wave is one component of such an ERP. The P3 amplitude is considered a marker for sensory processing and cognitive function, and a purported substitute indicator (i.e., endophenotype) for risk of alcoholism and other disinhibitory disorders.
